# Inverse Lomax-Rayleigh distribution with application

**DOI:** 10.1016/j.heliyon.2021.e08383

**Published:** 2021-11-18

**Authors:** Jamilu Yunusa Falgore, Muhammad Nazir Isah, Hussein Ahmad Abdulsalam

**Affiliations:** Department of Statistics, Ahmadu Bello University, Zaria, Nigeria

**Keywords:** Inverse Lomax G, Moments, Probability distribution, T-X approach, Inverse Lomax Rayleigh

## Abstract

In this paper, an extension of Rayleigh distribution called Inverse Lomax Rayleigh (ILR) is proposed by using the Inverse Lomax generator of [13]. Properties of ILR were derived. This includes the complete and incomplete moments, entropy, distribution of order statistics, and quantile function. A simulation study was presented to explore the properties of the estimates. This shows that they are unbiased, consistent, and efficient. An application to fatigue data shows the flexibility of ILR distribution, as it outperforms all the comparators with minimum values of all the measures.

## 1 Background

Using probability distributions to represent real-life situations is one of the most important tasks of a statistician. Modeling and interpreting lifetime data is essential in many practical situations, such as medical, actuarial science, engineering, and finance. In recent decades, this has prompted academics to focus on developing families of probability distributions.

Some of the recent families of distributions in the literature include Kumaraswamy Poisson G by [11], Zubair G by [3], Beta Poisson G by [15], Extended Exp G by [8], Inverse Lomax G by [13], Burr X Exponential G by [24], Odd Log-Logistic Lindley G by [7], Weibull Marshall-Olkin Lindley G by [2], Kumaraswamy-Odd Rayleigh-G by [14], Inverse Lomax Exponentiated G by [12], as well as Topp-Leone Odd Frechet G by [5], among others. Extension of probability distributions is a regular practice in the theory of statistics. Different strategies are proposed to generalize probability distributions in the literature. This is necessary so as the addition of parameter(s) will expand the adaptability of the models to catch the multifaceted nature of the data. Several generalized (or G) classes of distributions are available in the literature, but our main focus in this paper is to extend the Rayleigh distribution with the Inverse Lomax G family.

### 1.1 The Rayleigh distribution

The Cumulative distribution function *cdf* and probability density function *pdf* of Rayleigh distribution are given by

(1) $$G(x;\sigma )=1-e^{-\frac{x^{2}}{2\sigma^{2}}},\;x\in R,$$

and

(2) $$g(x;\sigma )=\frac{x}{\sigma^{2}}e^{-\frac{x^{2}}{2\sigma^{2}}},\;x\in R,$$

where σ>0.

The Rayleigh distribution has several applications, including life testing experiments, communication theory, technology, reliability analysis, applied statistics, medical testing, and clinical studies. With regard to this significance and the desire to give this distribution greater versatility, several researchers have proposed extensions to the Rayleigh distribution. This includes Odd Lindley-Rayleigh distribution by [16], Lomax-Rayleigh distribution by [4], Rayleigh-Rayleigh distribution by [6], an extension of Rayleigh distribution by [10], Weibull Rayleigh by [21], Transmuted Rayleigh by [19], New generalized Rayleigh distribution by [25], Generalized Rayleigh distribution by [17], among others.

## 2 Inverse Lomax family of distributions

Falgore and Doguwa [13], introduced the Inverse Lomax family of distribution by adopting T-X methodology by [9]. The *cdf* and *pdf* of the family have the following form

(3) $$F(x;\alpha ,\lambda ,\Delta )={\left(1+\frac{\alpha \overset{¯}{G}(x;\Delta )}{G(x;\Delta )}\right)}^{-\lambda },\;x\in R,$$

and

(4) $$f(x;\alpha ,\lambda ,\Delta )=\frac{\alpha \lambda g(x;\Delta )}{G{\left(x;\Delta \right)}^{2}}{\left(1+\frac{\alpha \overset{¯}{G}(x;\Delta )}{G(x;\Delta )}\right)}^{-\lambda -1},\;x\in R,$$

where α,λ>0, g(x;Δ) is the pdf of the baseline distribution and G(x;Δ) is the *cdf* of the baseline distribution, and $\overset{¯}{G}(x;\Delta )=1-G(x;\Delta )$, and *Δ* is a vector of parameter(s).

## 3 Inverse Lomax-Rayleigh distribution

By considering Rayleigh as baseline distribution, we have the *cdf* and *pdf* of the Inverse Lomax-Rayleigh distribution that follows from equations (3) and (4) given by

(5) $$F_{ILR}(x;\alpha ,\lambda ,\sigma )={\left[1+\alpha \left(\frac{e^{-\frac{x^{2}}{2\sigma^{2}}}}{1-e^{-\frac{x^{2}}{2\sigma^{2}}}}\right)\right]}^{-\lambda },\;x\in R$$

and

(6) $$f_{ILR}(x;\alpha ,\lambda ,\sigma )=\frac{\alpha \lambda xe^{-\frac{x^{2}}{2\sigma^{2}}}}{\sigma^{2}{\left(1-e^{-\frac{x^{2}}{2\sigma^{2}}}\right)}^{2}}{\left[1+\alpha \left(\frac{e^{-\frac{x^{2}}{2\sigma^{2}}}}{1-e^{-\frac{x^{2}}{2\sigma^{2}}}}\right)\right]}^{-\lambda -1},\;x\in R$$

where α,σ>0 are scale parameters & λ>0 is a shape parameter. The survival, hazard, and reverse hazard functions can be represented as

(7) $$S_{ILR}(x;;\alpha ,\lambda ,\sigma )=1-{\left[1+\alpha \left(\frac{e^{-\frac{x^{2}}{2\sigma^{2}}}}{1-e^{-\frac{x^{2}}{2\sigma^{2}}}}\right)\right]}^{-\lambda }$$

(8) $$h_{ILR}(x;;\alpha ,\lambda ,\sigma )=\frac{\alpha \lambda xe^{-\frac{x^{2}}{2\sigma^{2}}}{\left[1+\alpha \left(\frac{e^{-\frac{x^{2}}{2\sigma^{2}}}}{1-e^{-\frac{x^{2}}{2\sigma^{2}}}}\right)\right]}^{-\lambda -1}}{\sigma^{2}{\left(1-e^{-\frac{x^{2}}{2\sigma^{2}}}\right)}^{2}\left\{1-{\left[1+\alpha \left(\frac{e^{-\frac{x^{2}}{2\sigma^{2}}}}{1-e^{-\frac{x^{2}}{2\sigma^{2}}}}\right)\right]}^{-\lambda }\right\}}$$

(9) $$r_{ILR}(x;\alpha ,\lambda ,\sigma )=\frac{\alpha \lambda xe^{-\frac{x^{2}}{2\sigma^{2}}}}{\sigma^{2}{\left(1-e^{-\frac{x^{2}}{2\sigma^{2}}}\right)}^{2}}{\left[1+\alpha \left(\frac{e^{-\frac{x^{2}}{2\sigma^{2}}}}{1-e^{-\frac{x^{2}}{2\sigma^{2}}}}\right)\right]}^{-1}$$

**Validity Test** The pdf in equation (6) is valid. For a pdf to be valid, the $\int_{-\infty }^{\infty }f(x)dx$ must be 1. In this case,

(10) $$\int_{-\infty }^{\infty }f(x;\alpha ,\lambda ,\sigma )dx=\int_{0}^{\infty }\frac{\alpha \lambda xe^{-\frac{x^{2}}{2\sigma^{2}}}}{\sigma^{2}{\left(1-e^{-\frac{x^{2}}{2\sigma^{2}}}\right)}^{2}}{\left[1+\alpha \left(\frac{e^{-\frac{x^{2}}{2\sigma^{2}}}}{1-e^{-\frac{x^{2}}{2\sigma^{2}}}}\right)\right]}^{-\lambda -1}dx$$

which is the same as

$$\frac{\alpha \lambda }{\sigma^{2}}\int_{0}^{\infty }\frac{xe^{-\frac{x^{2}}{2\sigma^{2}}}}{{\left(1-e^{-\frac{x^{2}}{2\sigma^{2}}}\right)}^{2}}{\left[1+\alpha \left(\frac{e^{-\frac{x^{2}}{2\sigma^{2}}}}{1-e^{-\frac{x^{2}}{2\sigma^{2}}}}\right)\right]}^{-\lambda -1}dx,$$

$$\text{substituting}\;y=\alpha \left(\frac{e^{-\frac{x^{2}}{2\sigma^{2}}}}{1-e^{-\frac{x^{2}}{2\sigma^{2}}}}\right),$$

we arrived at

$$\lambda \int_{0}^{\infty }{\left(1+y\right)}^{-1-\lambda }dy=1$$

## 4 Quantile function

The quantile function of ILD can be derived by inverting equation (5) as follows

Let

$$U={\left[1+\alpha \left(\frac{e^{-\frac{x^{2}}{2\sigma^{2}}}}{1-e^{-\frac{x^{2}}{2\sigma^{2}}}}\right)\right]}^{-\lambda },$$

$$\frac{U^{-\frac{1}{\lambda }}-1}{\alpha }=\left(\frac{e^{-\frac{x^{2}}{2\sigma^{2}}}}{1-e^{-\frac{x^{2}}{2\sigma^{2}}}}\right),$$

$$U^{-\frac{1}{\lambda }}-U^{-\frac{1}{\lambda }}e^{-\frac{x^{2}}{2\sigma^{2}}}-1+e^{-\frac{x^{2}}{2\sigma^{2}}}=\alpha e^{-\frac{x^{2}}{2\sigma^{2}}}$$

$$e^{-\frac{x^{2}}{2\sigma^{2}}}=\frac{1-U^{-\frac{1}{\lambda }}}{1-\alpha -U^{-\frac{1}{\lambda }}}$$

by taking log of both sides and some simplifications, we have the quantile function as

(11) $$x={\left[-2\sigma^{2}log\left(\frac{1-U^{-\frac{1}{\lambda }}}{1-\alpha -U^{-\frac{1}{\lambda }}}\right)\right]}^{\frac{1}{2}}$$

## 5 Mixture form

The pdf in equation (6) can be re-written in closed form. This form can be used in deriving basic properties such as moments, entropies, and distribution of order statistics.

$$f(x;\alpha ,\lambda ,\sigma )=\frac{\alpha \lambda xe^{-\frac{x^{2}}{2\sigma^{2}}}}{\sigma^{2}{\left(1-e^{-\frac{x^{2}}{2\sigma^{2}}}\right)}^{2}}{\left[1+\alpha \left(\frac{e^{-\frac{x^{2}}{2\sigma^{2}}}}{1-e^{-\frac{x^{2}}{2\sigma^{2}}}}\right)\right]}^{-\lambda -1}$$

recall this identity

(12) $${\left(1+z\right)}^{-n}=\sum_{i=1}^{n}\frac{{\left(-1\right)}^{i}\Gamma (n+i)}{i!\Gamma (n)}z^{i}\Rightarrow {\left[1+\alpha \left(\frac{e^{-\frac{x^{2}}{2\sigma^{2}}}}{1-e^{-\frac{x^{2}}{2\sigma^{2}}}}\right)\right]}^{-(\lambda +1)}=\sum_{i=1}^{n}\frac{{\left(-1\right)}^{i}\Gamma (\lambda +1+i)}{i!\Gamma (\lambda +1)}\alpha^{i}{\left(\frac{e^{-\frac{x^{2}}{2\sigma^{2}}}}{1-e^{-\frac{x^{2}}{2\sigma^{2}}}}\right)}^{i}$$

after simplifications and replacement, we have

$$f(x;\alpha ,\lambda ,\sigma )=\frac{\alpha^{i+1}\lambda x}{\sigma^{2}}\sum_{i=1}^{n}\frac{{\left(-1\right)}^{i}\Gamma_{\left(\lambda +1+i\right)}}{i!\Gamma (\lambda +1)}{\left(1-e^{-\frac{x^{2}}{2\sigma^{2}}}\right)}^{-2-i}{\left(e^{-\frac{x^{2}}{2\sigma^{2}}}\right)}^{1+i}=\frac{\alpha^{i+1}\lambda x}{\sigma^{2}}\sum_{i=1}^{n}\frac{{\left(-1\right)}^{i}\Gamma_{\left(\lambda +1+i\right)}}{i!\Gamma (\lambda +1)}{\left(1-e^{-\frac{x^{2}}{2\sigma^{2}}}\right)}^{-(2+i)}e^{-\frac{x^{2}}{2\sigma^{2}}(1+i)},$$

another identity is

(13) $${\left(1-z\right)}^{-n}=\sum_{j=1}^{\infty }\frac{\Gamma (n+j)}{j!\Gamma (n)}z^{j}\Rightarrow {\left(1-e^{-\frac{x^{2}}{2\sigma^{2}}}\right)}^{-(2+i)}=\sum_{j=1}^{\infty }\frac{\Gamma (2+i+j)}{j!\Gamma (2+i)}{\left(e^{-\frac{x^{2}}{2\sigma^{2}}}\right)}^{j}=\sum_{j=1}^{\infty }\frac{\Gamma (2+i+j)}{j!\Gamma (2+i)}e^{-\frac{x^{2}}{2\sigma^{2}}j}$$

after replacing the above quantity back, it becomes

(14) $$f(x;\alpha ,\lambda ,\sigma )=\frac{\alpha^{i+1}\lambda x}{\sigma^{2}\Gamma (\lambda +1)}\sum_{i=1}^{n}\sum_{j=1}^{\infty }\frac{{\left(-1\right)}^{i}\Gamma (\lambda +1+i)\Gamma (2+i+j)}{i!j!\Gamma (2+i)}{\left(e^{-\frac{x^{2}}{2\sigma^{2}}}\right)}^{1+i+j}=\frac{\alpha \lambda x}{\sigma^{2}\Gamma (\lambda +1)}\sum_{i=1}^{n}\sum_{j=1}^{\infty }\alpha^{i}\frac{{\left(-1\right)}^{i}\Gamma (\lambda +1+i)\Gamma (2+i+j)}{i!j!\Gamma (2+i)}e^{-\frac{x^{2}}{2\sigma^{2}}(1+i+j)}=\frac{\alpha \lambda }{\sigma^{2}\Gamma (\lambda +1)}\sum_{ij}\xi_{ij}xe^{-\frac{x^{2}}{2\sigma^{2}}(1+i+j)},$$

where

(15) $$\xi_{ij}=\alpha^{i}\frac{{\left(-1\right)}^{i}\Gamma (\lambda +1+i)\Gamma (2+i+j)}{i!j!\Gamma (2+i)}$$

## 6 Moment and moment generating function

### 6.1 Moment

The moments of the ILR distribution can be given in terms of the mixture representations discussed in section 5.

$$f(x;\alpha ,\lambda ,\sigma )=\frac{\alpha \lambda }{\sigma^{2}\Gamma (\lambda +1)}\sum_{ij}\xi_{ij}xe^{-\frac{x^{2}}{2\sigma^{2}}(1+i+j)}$$

(16) $$\mu_{r}^{\prime }=\int_{-\infty }^{\infty }x^{r}f(x)dx,$$

(17) $$=\int_{0}^{\infty }x^{r}\frac{\alpha \lambda }{\sigma^{2}\Gamma (\lambda +1)}\sum_{ij}\xi_{ij}xe^{-\frac{x^{2}}{2\sigma^{2}}(1+i+j)}dx,=\frac{\alpha \lambda }{\sigma^{2}\Gamma (\lambda +1)}\sum_{ij}\xi_{ij}\int_{0}^{\infty }x^{r+1}e^{-\frac{x^{2}}{2\sigma^{2}}(1+i+j)}dx,$$

substituting $y=\frac{x^{2}}{2\sigma^{2}}(1+i+j)$, we arrived at

(18) $$\mu_{r}^{\prime }=\frac{\alpha \lambda }{\sigma^{2}\Gamma (\lambda +1)}\sum_{ij}\xi_{ij}\int_{0}^{\infty }{\left(\frac{\sigma 2^{\frac{1}{2}}y^{\frac{1}{2}}}{{\left(1+i+j\right)}^{\frac{1}{2}}}\right)}^{r}e^{-y}\frac{\sigma^{2}dy}{1+i+j}=\frac{2^{\frac{r}{2}}\alpha \lambda \sigma^{r}}{\Gamma (\lambda +1)}\sum_{ij}\frac{\xi_{ij}}{{\left(1+i+j\right)}^{\frac{r+2}{2}}}\int_{0}^{\infty }y^{\frac{r}{2}}e^{-y}dy,\mu_{r}^{\prime }=\frac{2^{\frac{r}{2}}\alpha \lambda \sigma^{r}}{\Gamma (\lambda +1)}\sum_{ij}\frac{\xi_{ij}}{{\left(1+i+j\right)}^{\frac{r+2}{2}}}\Gamma (\frac{r+2}{2})$$

### 6.2 Moment generating function

The moment generating function of ILR distribution can be given in terms of the moments as shown below

(19) $$M(t)=E(e^{tx})=\int_{-\infty }^{\infty }e^{tx}f(x)dx,$$

(20) $$=\sum_{r=0}^{\infty }\frac{t^{r}}{r!}\int_{0}^{\infty }x^{r}f(x)dx,=\sum_{r=0}^{\infty }\frac{t^{r}}{r!}\mu_{r}^{\prime }.$$

## 7 Renyi entropy of ILR distribution

The entropy considered here is the Renyi entropy by [23]. The Renyi entropy for the ILR random variable is given by

(21) $$I_{R}(c)=\frac{1}{1-c}log\int_{0}^{\infty }f_{c}(x)dx,\;c\geq 0\;\&\;c\neq 1$$

$$f^{c}(x;\alpha ,\lambda ,\sigma )={\left[\frac{\alpha \lambda }{\sigma^{2}\Gamma_{\left(\lambda +1\right)}}\sum_{ij}\xi_{ij}xe^{-\frac{x^{2}}{2\sigma^{2}}(1+i+j)}\right]}^{c},=\frac{\alpha^{c}\lambda^{c}}{\sigma^{2c}{\left(\Gamma (\lambda +1)\right)}^{c}}\sum_{ij}\xi_{ij}^{c}x^{c}e^{-\frac{cx^{2}}{2\sigma^{2}}(1+i+j)},$$

by replacing back, we have

$$I_{R}(c)=\frac{1}{1-c}log\int_{0}^{\infty }\frac{\alpha^{c}\lambda^{c}}{\sigma^{2c}{\left(\Gamma (\lambda +1)\right)}^{c}}\sum_{ij}\xi_{ij}^{c}x^{c}e^{-\frac{cx^{2}}{2\sigma^{2}}(1+i+j)}dx,=\frac{1}{1-c}log\frac{\alpha^{c}\lambda^{c}}{\sigma^{2c}{\left(\Gamma (\lambda +1)\right)}^{c}}\sum_{ij}\xi_{ij}^{c}\int_{0}^{\infty }x^{c}e^{-\frac{cx^{2}}{2\sigma^{2}}(1+i+j)}dx$$

substituting $f_{1}=\frac{cx^{2}}{2\sigma^{2}}(1+i+j)$, gives

(22) $$I_{R}(c)=\frac{1}{1-c}log\left[\frac{2^{\frac{c}{2}-1}\alpha^{c}\lambda^{c}\sigma^{\left(2-c\right)}}{{\left(\Gamma (\lambda +1)\right)}^{c}c^{\frac{c}{2}}}\sum_{ij}\frac{\xi_{ij}^{c}}{{\left(1+i+j\right)}^{\frac{c}{2}}}\int_{0}^{\infty }f_{1}^{\frac{c}{2}-1}e^{-f_{1}}df_{1}\right],=\frac{1}{1-c}log\left[\frac{2^{\frac{c}{2}-1}\alpha^{c}\lambda^{c}\sigma^{\left(2-c\right)}}{{\left(\Gamma (\lambda +1)\right)}^{c}c^{\frac{c}{2}}}\sum_{ij}\frac{\xi_{ij}^{c}}{{\left(1+i+j\right)}^{\frac{c}{2}}}\Gamma (\frac{c}{2})\right]$$

## 8 Pdf of the $k^{th}$ order statistics for the ILR distribution

Order statistics are important in statistical theory especially in the theory of extreme value. The pdf of the $k^{th}$ order statistics of the ILR is derived here

(23) $$f_{k,n}(x)=\frac{n!}{(k-1)!(n-k)!}f(x){\left[F(x)\right]}^{k-1}{\left[1-F(x)\right]}^{n-k},$$

for simplicity, we use

$${\left[1-F(x)\right]}^{n-k}=\sum_{a=0}^{\infty }\left(\begin{array}{c} n-k\\ a \end{array}\right){\left(-1\right)}^{a}{\left[F(x)\right]}^{a}.$$

Now,

(24) $$f_{k,n}(x)=\frac{n!}{(k-1)!(n-k)!}f(x)\sum_{a=0}^{\infty }\left(\begin{array}{c} n-k\\ a \end{array}\right){\left(-1\right)}^{a}{\left[F(x)\right]}^{a+k-1}=\frac{n!}{(k-1)!(n-k)!}\frac{\alpha \lambda }{\sigma^{2}\Gamma (\lambda +1)}\sum_{ij}\xi_{ij}xe^{-\frac{x^{2}}{2\sigma^{2}}(1+i+j)}\times \sum_{a=0}^{\infty }\left(\begin{array}{c} n-k\\ a \end{array}\right){\left(-1\right)}^{a}{\left[1+\alpha \left(\frac{e^{-\frac{x^{2}}{2\sigma^{2}}}}{1-e^{-\frac{x^{2}}{2\sigma^{2}}}}\right)\right]}^{-\lambda (a+k-1)},=\frac{n!}{(k-1)!}\frac{\alpha \lambda }{\sigma^{2}\Gamma (\lambda +1)}\sum_{ij}\xi_{ij}xe^{-\frac{x^{2}}{2\sigma^{2}}(1+i+j)}\times \sum_{a=0}^{\infty }\frac{{\left(-1\right)}^{a}}{(n-k-a)!a!}{\left[1+\alpha \left(\frac{e^{-\frac{x^{2}}{2\sigma^{2}}}}{1-e^{-\frac{x^{2}}{2\sigma^{2}}}}\right)\right]}^{-\lambda (a+k-1)}$$

### 8.1 Largest order statistic

Here k=n

(25) $$f_{n,n}(x)=\frac{n\alpha \lambda }{\sigma^{2}\Gamma (\lambda +1)}\sum_{ij}\xi_{ij}xe^{-\frac{x^{2}}{2\sigma^{2}}(1+i+j)}\times \sum_{a=0}^{\infty }\frac{{\left(-1\right)}^{a}}{(-a)!a!}{\left[1+\alpha \left(\frac{e^{-\frac{x^{2}}{2\sigma^{2}}}}{1-e^{-\frac{x^{2}}{2\sigma^{2}}}}\right)\right]}^{-\lambda (a+n-1)}$$

### 8.2 Smallest order statistic

Here k=1

(26) $$f_{1,n}(x)=\frac{n!\alpha \lambda }{\sigma^{2}\Gamma (\lambda +1)}\sum_{ij}\xi_{ij}xe^{-\frac{x^{2}}{2\sigma^{2}}(1+i+j)}\times \frac{{\left(-1\right)}^{a}}{(n-1-a)!a!}{\left[1+\alpha \left(\frac{e^{-\frac{x^{2}}{2\sigma^{2}}}}{1-e^{-\frac{x^{2}}{2\sigma^{2}}}}\right)\right]}^{-a\lambda }$$

## 9 $s^{th}$ Incomplete moment of X

Incomplete moments play a significance role in computing measures of statistical theory. The $s^{th}$ incomplete moments $m_{s}(y)$ of the ILR distribution are

(27) $$m_{s}(y)=\int_{0}^{y}x^{s}f(x)dx$$

$$m_{s}(y)=\int_{0}^{y}x^{s}\frac{\alpha \lambda }{\sigma^{2}\Gamma (\lambda +1)}\sum_{ij}\xi_{ij}xe^{-\frac{x^{2}}{2\sigma^{2}}(1+i+j)}dx,=\frac{\alpha \lambda }{\sigma^{2}\Gamma (\lambda +1)}\sum_{ij}\xi_{ij}\int_{0}^{y}x^{s+1}e^{-\frac{x^{2}}{2\sigma^{2}}(1+i+j)}dx,$$

substituting $w=\frac{x^{2}}{2\sigma^{2}}(1+i+j)$, we arrived at

(28) $$m_{s}(y)=\frac{\alpha \lambda \sigma^{s}}{\Gamma (\lambda +1)}\sum_{ij}\xi_{ij}\frac{2^{\frac{s}{2}}}{{\left(1+i+j\right)}^{\frac{s}{2}+1}}\int_{0}^{\frac{y^{2}(1+i+j)}{2\sigma^{2}}}w^{\frac{s}{2}}e^{-w}dw,=\frac{\alpha \lambda \sigma^{s}}{\Gamma (\lambda +1)}\sum_{ij}\xi_{ij}\frac{2^{\frac{s}{2}}}{{\left(1+i+j\right)}^{\frac{s}{2}+1}}\gamma \left(\frac{y^{2}(1+i+j)}{2\sigma^{2}},\frac{s}{2}+1\right)$$

## 10 Inequality measures

Lorenz and Bonferroni curves are used in various areas like reliability studies, econometrics, and insurance.

### 10.1 Lorenz curve

Lorenz curve for ILR distribution can be given as

(29) $$L_{G}(x)=\frac{1}{\mu }\int_{0}^{y}xf(x)dx,$$

where $\int_{0}^{y}xf(x)dx$, is the $1^{st}$ incomplete moment, $L_{G}(x)=\frac{\alpha \lambda \sigma }{\Gamma (\lambda +1)}\sum_{ij}\xi_{ij}\times \frac{2^{\frac{1}{2}}}{{\left(1+i+j\right)}^{\frac{3}{2}}}\gamma \left(\frac{y^{2}(1+i+j)}{2\sigma^{2}},\frac{3}{2}\right)$

(30) $$∴L_{G}(x)=\frac{\alpha \lambda \sigma }{\mu \Gamma (\lambda +1)}\sum_{ij}\xi_{ij}\frac{2^{\frac{1}{2}}}{{\left(1+i+j\right)}^{\frac{3}{2}}}\gamma \left(\frac{y^{2}(1+i+j)}{2\sigma^{2}},\frac{3}{2}\right)$$

### 10.2 Bonferoni curve

The Bonferoni curve for the ILR distribution can be given as

(31) $$B_{G}(x)=\frac{L_{G}(x)}{G(x)},$$

(32) $$=\frac{\alpha \lambda \sigma }{G(x)\mu \Gamma (\lambda +1)}\sum_{ij}\xi_{ij}\frac{2^{\frac{1}{2}}}{{\left(1+i+j\right)}^{\frac{3}{2}}}\gamma \left(\frac{y^{2}(1+i+j)}{2\sigma^{2}},\frac{3}{2}\right)$$

## 11 The maximum likelihood estimation

Let $x_{1},x_{2},x_{3},\ldots ,x_{n}$ be the observed values of n observations independently drawn from the ILR distribution with parameter vector *θ*, $\theta ={\left(\alpha ,\lambda ,\sigma \right)}^{T}$. Then,

(33) $$f(x;\theta )=\frac{\alpha \lambda xe^{-\frac{x^{2}}{2\sigma^{2}}}}{\sigma^{2}{\left(1-e^{-\frac{x^{2}}{2\sigma^{2}}}\right)}^{2}}{\left[1+\alpha \left(\frac{e^{-\frac{x^{2}}{2\sigma^{2}}}}{1-e^{-\frac{x^{2}}{2\sigma^{2}}}}\right)\right]}^{-\lambda -1}.$$

The log-likelihood (ll) function for *θ* denoted by l(θ) can be expressed as

(34) $$l(\theta )=nlog\left(\frac{\alpha \lambda }{\sigma^{2}}\right)+\sum_{i=1}^{n}logx_{i}-\frac{\sum_{i=1}^{n}x_{i}^{2}}{2\sigma^{2}}-2\sum_{i=1}^{n}log\left(1-e\frac{-x_{i}^{2}}{2\sigma^{2}}\right)-(\lambda +1)\sum_{i=1}^{n}log\left[1+\alpha \left(\frac{e^{-\frac{x_{i}^{2}}{2\sigma^{2}}}}{1-e^{-\frac{x_{i}^{2}}{2\sigma^{2}}}}\right)\right]$$

haven taken the partial derivatives of Equation (34) with respect to *α*, *λ*, and *σ* we derived U(θ) i.e. the Score Vector components are as follows

(35) $$\frac{\partial l}{\partial \alpha }=\frac{n}{\alpha }-(\alpha l)\sum_{i=1}^{n}\left[\frac{\left(\frac{e^{-\frac{x_{i}^{2}}{2\sigma^{2}}}}{1-e^{-\frac{x_{i}^{2}}{2\sigma^{2}}}}\right)}{\left[1+\alpha \left(\frac{e^{-\frac{x_{i}^{2}}{2\sigma^{2}}}}{1-e^{-\frac{x_{i}^{2}}{2\sigma^{2}}}}\right)\right]}\right]$$

(36) $$\frac{\partial l}{\partial \lambda }=\frac{n}{\lambda }-\sum_{i=1}^{n}log\left[1+\alpha \left(\frac{e^{-\frac{x_{i}^{2}}{2\sigma^{2}}}}{1-e^{-\frac{x_{i}^{2}}{2\sigma^{2}}}}\right)\right]$$

(37) $$\frac{\partial l}{\partial \sigma }=-\frac{2n}{\sigma }+\frac{\sum_{i=1}^{n}x_{i}^{2}}{\sigma^{3}}-\frac{2}{\sigma^{3}}\sum_{i=1}^{n}\left[\frac{x_{i}^{2}e^{-\frac{x_{i}^{2}}{2\sigma^{2}}}}{1-e^{-\frac{x_{i}^{2}}{2\sigma^{2}}}}\right]-\frac{\alpha (\lambda +1)}{\sigma^{3}}\sum_{i=1}^{n}\left[\frac{x_{i}^{2}e^{-\frac{x_{i}^{2}}{2\sigma^{2}}}}{{\left(1-e^{-\frac{x_{i}^{2}}{2\sigma^{2}}}\right)}^{2}\left[1+\alpha \left(\frac{e^{-\frac{x_{i}^{2}}{2\sigma^{2}}}}{1-e^{-\frac{x_{i}^{2}}{2\sigma^{2}}}}\right)\right]}\right]$$

Setting Equations (35), (36), and (37) to zero and also solving simultaneously yields the MLE $\left(\overset{ˆ}{\theta })=(\overset{ˆ}{\alpha },\overset{ˆ}{\lambda },\overset{ˆ}{\sigma }\right)$ of *θ*. However, these equations can not be solved analytically. Therefore, statistical software can be employed to solve the equations numerically through iterative methods.

## 12 Simulation

In this section, a Monte Carlo simulation analysis is performed and the findings are presented to demonstrate the performance of the estimates at different true parameter values. We set the true parameter values as (α=0.6, λ=0.5, σ=0.3). The numerical study is described as follows:

(i). For true parameter values i.e. $\theta ={\left(\lambda ,\sigma ,\alpha \right)}^{T}$, we simulated a random sample of size n from the ILR distribution using the quantile function defined in Equation (11).

(ii). We then Estimate the parameters of the ILR distribution from the sample using method of maximum likelihood.

(iii). We conduct N=1,000 replications of steps (i) and (ii).

(iv). For each of the three (3) estimated parameters of the ILR, from the N replicates, we compute the mean estimate, Bias, and MSE. The statistics are given by

(38) $$\overset{ˆ}{\theta }=\frac{1}{N}\sum_{i=1}^{N}\overset{ˆ}{\theta_{i}},\;Bias(\overset{ˆ}{\zeta })=\overset{ˆ}{\theta }-\theta ,\;var(\overset{ˆ}{\theta })=\sum_{i=1}^{N}\frac{{\left(\overset{ˆ}{\theta_{i}}-\overset{ˆ}{\theta }\right)}^{2}}{N}\;MSE(\overset{ˆ}{\theta })=var(\overset{ˆ}{\theta })+{\left(Bias(\overset{ˆ}{\theta })\right)}^{2}$$

where the vector of estimated parameters $\overset{ˆ}{\theta_{i}}$ is the maximum likelihood estimate for each iteration (n=30,75,150,300,500,1,000).

The simulation results are presented in Table 1. The simulation study has shown that irrespective of the parameter values chosen, the Bias and MSE of the parameter estimate decay as the sample size n increases. Thus, the larger the sample size, the more consistent are the estimates of the parameters. The estimates are good as they approach the true parameter values as the sample size increases.

**Table 1:** The Estimate, Bias, and MSE for the parameters of ILR distribution.

n	Properties	*α* = 0.6	*λ* = 0.5	*σ* = 0.3
30	Est.	0.6014	0.5003	0.1119
	Bias	0.0014	0.0003	-0.1881
	MSE	0.0009	0.0002	0.0438

75	Est.	0.6013	0.5004	0.1357
	Bias	0.0013	0.0004	-0.1643
	MSE	0.0009	7.4e-05	0.0389

150	Est.	0.6003	0.5006	0.1419
	Bias	0.0003	0.0006	-0.1581
	MSE	1.7e-07	6.01e-07	0.0396

300	Est.	0.6003	0.5006	0.1485
	Bias	0.0003	0.0006	-0.1515
	MSE	1.72e-07	5.81e-07	0.0399

500	Est.	0.6012	0.5005	0.1531
	Bias	0.0012	0.0005	-0.1469
	MSE	0.0008	1.14e-05	0.0398

1000	Est.	0.6003	0.5006	0.1637
	Bias	0.0003	0.0006	-0.1363
	MSE	1.61e-07	5.47e-07	0.0380

## 13 Application

A demonstration of the applicability of the ILR was demonstrated using Fatigue data as in [1]. The summary of the data is as follows: n=76, minimum = 0.0251, maximum = 9.9096, mean = 1.9592, mode = 1.5, median = 1.7362, variance = 2.4774, skewness = 1.9796, as well as kurtosis = 5.1608. Table 2 summarizes the comparators with their references. They are: Transmuted Generalized Rayleigh (TGR) distribution, Weibull-Rayleigh (WR) distribution, Type II Topp-Leone Generalized Inverse Rayleigh (TLR) distribution, and Rayleigh distribution, respectively. Table 4 presents the criteria for selecting fitted models. A package in R software called Adequacy Model by [18] was used in the analysis.

**Table 2:** Competing Models with ILR distribution.

Models	References
TGR	[20]
WR	[21]
TLR	[26]
R	[22]

The Maximum Likelihood estimates with the standard errors in (parentheses) for the ILR distribution are presented in Table 3. These are the estimated values of the parameters of the ILR distribution based on the Fatigue data set. The standard errors can be used to compute the confidence interval for drawing inferences. In Table 4, ILR seems to be the best with large P-Value and smaller AIC, CAIC, BIC, HQIC, -ll, and KS, respectively. This shows that the proposed ILR distribution fits the Fatigue data better than the compared distributions.

**Table 3:** Maximum Likelihood Estimates (with standard errors) for the ILR distributions and other comparators.

Models	MLEs		
	$\overset{ˆ}{\alpha }$	$\overset{ˆ}{\lambda }$	$\overset{ˆ}{\delta }$
ILR	0.1816	0.7525	3.2501
	(0.1139)	(0.1389)	(0.7495)
TGR	0.6242	0.2649	0.7179
	(0.0765)	(0.0389)	(0.2284)
WR	7.9	0.6336	0.0152
	(3.0821)	(0.0529)	(0.0079)
T2R	1.3066	0.0028	0.1825
	(0.646)	(0.0025)	(0.0227)
R			1.7725
			(0.1017)

**Table 4:** Measurement criteria for the ILR and other comparators.

Models	AIC	CAIC	BIC	HQIC	-ll	P-Value	K.S
ILR	249.1795	249.1717	256.1717	251.9739	121.5898	5.43E-01	0.0897
TGR	251.6984	252.0317	258.6906	254.4928	122.8486	2.27E-01	0.1174
WR	252.6481	252.9815	259.6403	255.4426	123.3241	1.37E-01	0.1306
TLR	388.2653	388.5986	395.2575	391.0597	191.1327	3.01E-01	0.2175
R	276.6394	276.6935	278.9702	277.5709	137.3197	2.90E-03	0.2043

## 14 Concluding remarks

**Figure 1:**
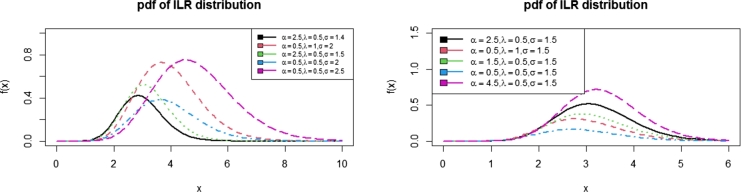
Plots of the ILR density function.

**Figure 2:**
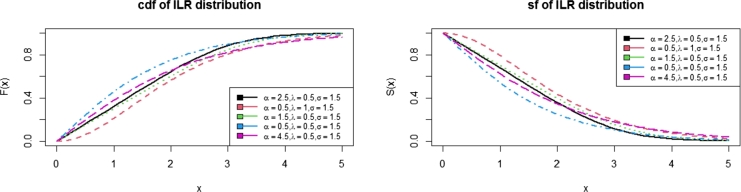
Plots of the ILR cdf and sf functions.

**Figure 3:**
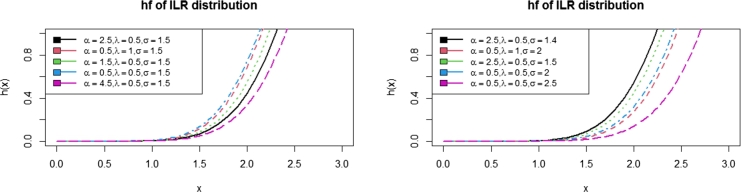
Plots of the ILR hazard function.

**Figure 4:**
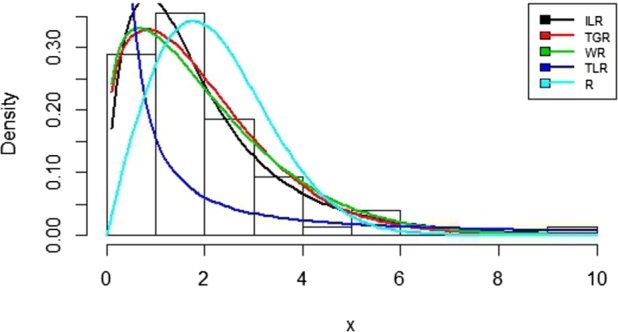
Fitted Densities for the Fatique data set.

In this paper, a new sub-model of the Inverse Lomax G family of distributions was proposed. It is called Inverse Lomax Rayleigh (ILR) distribution. Some of the properties of ILR distribution were presented. Figure 1, Figure 2, Figure 3 show the pdfs, hazards, cdf, and survival functions, respectively. This indicates that the ILR distribution can take symmetric and asymmetric shapes depending on the values of the parameters. To test the proposed distribution, a simulation study was conducted by setting the initial values of the parameters as (α=0.6, λ=0.5, σ=0.3) for 1,000 iterations. Furthermore, the ILR distribution was fitted to a Fatigue data set alongside some other distributions in the literature. Based on the results in Table 4, the proposed ILR distribution seems to be the best. Fig. 4 also indicated that the ILR distribution fitted the data best than the other comparators.

## Declarations

### Author contribution statement

M. I. Nazir: Conceived and designed the experiments. H. A. Abdulsalam: Performed the experiments; Wrote the paper. J. Y. Falgore: Analyzed and interpreted the data; Wrote the paper.

### Funding statement

This research did not receive any specific grant from funding agencies in the public, commercial, or not-for-profit sectors.

### Data availability statement

Data included in article/supplementary material/referenced in article.

### Declaration of interests statement

The authors declare no conflict of interest.

### Additional information

No additional information is available for this paper.
